# Evaluation of invasive and non-invasive methods for the diagnosis of *Helicobacter pylori* infection in symptomatic children and adolescents

**DOI:** 10.1590/S1516-31802001000200006

**Published:** 2001-03-02

**Authors:** Silvio Kazuo Ogata, Elisabete Kawakami, Francy Reis Silva Patrício, Margareth Zabeu Pedroso, Antonio Mario Santos

**Keywords:** Children and adolescents, Helicobacter pylori, Diagnostic methods, Crianças e adolescentes, Helicobacter pylori, Métodos diagnósticos, ABREVIAÇÕES: Hp - Helicobacter pylori

## Abstract

**CONTEXT:**

Multiple diagnostic methods are available for the detection of *Helicobacter pylori* infection, but at present no single one can be used as the gold standard.

**OBJECTIVE:**

The aim of this study was to evaluate the diagnostic accuracy of 3 invasive and 2 non-invasive methods for detection of *Helicobacter pylori* infection in symptomatic children and adolescents.

**DESIGN:**

Prospectivecohort study

**SETTING:**

Peptic Disease outpatients service, Discipline of Pediatric Gastroenterology, Universidade Federal de São Paulo / Escola Paulista de Medicina.

**PATIENTS:**

Forty-seven patients who underwent endoscopy because of dyspeptic symptoms.

**DIAGNOSTIC METHODS:**

Endoscopy with gastric biopsies for 3 invasive (rapid urease test, histology and culture) and 2 non-invasive methods (a commercial ELISA serology and ^13^carbon urea breath test - isotope ratio mass spectrometry) for detection of *Helicobacter pylori* infection.

**MAIN MEASUREMENTS:**

Sensitivity, specificity, positive and negative predictive values of each method and agreement and disagreement rates between the methods.

**RESULTS:**

Forty-seven patients [mean age, 11y9mo (SD 2y10mo), 27 female and 20 male]; 62% of them were *Helicobacter pylori*-positive. All methods agreed in 61%, and were negative in 21% and positive in 40%. The greatest concordance between 2 methods occurred between the invasive methods: histology and rapid urease test (89.6%) and histology and culture (87.5%). The greatest sensitivity, considering *Helicobacter pylori*-positive cases, for any combination of 3 or more tests, was achieved by the rapid urease test (S=100%), followed by histology, serology and ^13^carbon-urea breath test (S=93.1%) and lastly by culture (S=79.3%). The highest specificity was obtained by histology (100%) and culture (100%), followed by the rapid urease test (84.2%), serology (78.9%) and ^13^carbon-urea breath test (78.9%).

**CONCLUSIONS:**

Our results suggest that among invasive methods, an association between the rapid urease test and histology constituted the best choice for the detection of *Helicobacter pylori* infection. If results of histology and the rapid urease test are different, serology may be recommended.

## RESUMO

**CONTEXTO:**

Vários métodos diagnósticos estão disponíveis para a detecção da infecção por *Helicobacter pylori* (Hp), porém, até o presente momento, não há um teste que possa ser utilizado isoladamente como padrão-ouro.

**OBJETIVO:**

Avaliar a acurácia de três métodos invasivos e dois não-invasivos na detecção da infecção por Hp em crianças e adolescentes sintomáticos.

**TIPO DE ESTUDO:**

Estudo coorte prospectivo.

**LOCAL:**

Ambulatório de Doença Péptica, Disciplina de Gastroenterologia Pediátrica, Universidade Federal de São Paulo / Escola Paulista de Medicina.

**PACIENTES:**

47 pacientes sintomáticos que realizaram exame endoscópico devido a sintomas dispépticos.

**MÉTODOS DIAGNÓSTICOS:**

Exame endoscópico com biopsias gástricas para três métodos invasivos (teste rápido da urease, histologia e cultura) e dois métodos não-invasivos (teste sorológico ELISA industrializado e teste respiratório com uréia marcada com Carbono^13^).

**VARIÁVEIS ESTUDADAS:**

Sensibilidade, especificidade, valor preditivo positivo e negativo de cada método e taxas de concordância e discordância entre os métodos.

**RESULTADOS:**

47 pacientes [idade média de 11a9m (DP 2a10m), 27 do sexo feminino e 20 do masculino], 62% deles com infecção por Hp. Todos os 5 métodos concordaram em 61%, sendo negativo em 21% e positivo em 40%. As maiores concordâncias entre dois métodos ocorreram entre os métodos invasivos: histologia e teste rápido da urease (89,6%) e entre a histologia e cultura (87,5%). A maior sensibilidade, considerando como Hp positivo, qualquer combinação de 3 ou mais testes, foi encontrada no teste rápido da urease (S=100%), seguido pela histologia, sorologia e o teste respiratório com uréia marcada com Carbono^13^ (S=93,1%) e por fim a cultura (S=79,3%). A maior especificidade foi obtida pela histologia e cultura (100%), seguidos pelo teste rápido da urease (84,2%), sorologia (78,9%) e teste respiratório com uréia marcada com Carbono^13^ (78,9%).

**CONCLUSÕES:**

Nossos resultados sugerem que, entre os métodos invasivos, a associação do teste rápido da urease e histologia constituem a melhor escolha para a detecção da infecção por Hp. Se os resultados da histologia e do teste rápido da urease forem discordantes é recomendada a sorologia.

## INTRODUCTION

In developing countries, the *Helicobacter pylori* prevalence in asymptomatic children is higher than in developed countries^1-3^ and in symptomatic children the infection rates vary from 45 to 82%.^4,5-8^ Nowadays, there is general consensus that *Helicobacter pylori* infection is the main etiological factor of primary gastritis in children and adults^9-12^ and a significant correlation between *Helicobacter pylori*-associated gastritis and peptic ulcer disease has been found, especially with duodenal ulcer^10,13,14^. Gastric cancer^4,15^ and lymphoproliferative gastric diseases, especially Malt lymphoma, also have been correlated with *Helicobacter pylori* infection.^16,17^ Recently, refractory iron-deficiency anemia with correction after Hp eradication has been re- ported.^18,19^

A reliable test to detect this infection is crucial, but none of the tests available is suitable for all situations, each having its own drawbacks and pitfalls.^20^ Invasive tests have been considerate the gold standard, but biopsy-based methods may suffer from sampling error, because of the patchy nature of the in-fection^20,21^ and low concentration of bacteria in fragments.^22^ Culturing has low sensitivity, and so no single test can be used as the gold standard^23^ and the tendency has been to use a combination of tests^20^ in adult and pediatric studies.^28,29^ Non-invasive tests are easier to accomplish but need appropriate equipment and validation of methods for each region, population and age.^28,29^ In Brazil, the *Helicobacter pylori* infection has high prevalence but there are no pediatric studies on the accuracy of invasive and non-invasive methods to diagnose *Helicobacter pylori* infection. And these methods need to be validated because of the high infection prevalence in our population.

The aim of this study was to evaluate the accuracy of 5 methods for diagnosis of *Helicobacter pylori* infection, 3 invasive (rapid urease test, histology and bacterial culture) and 2 non-invasive methods (serologic test and urea breath test).

## METHODS

The Medical Ethics Committee of our university approved the study and informed consent was obtained from the person responsible for each patient.

From March 1997 to October 1998, 47 outpatients with dyspeptic symptoms, who underwent diagnostic upper GI endoscopy with gastric biopsies, were prospectively evaluated. Patients with chronic extra-digestive or immunosuppressive disease and patients using immunosuppressor or chemotherapy drugs, anti-inflammatory drugs, H2 receptor antagonist, antimicrobial and/or nitroimidazole and/ or bismuth compounds, for least 3 months prior to the examination, were excluded.

### Invasive methods

Endoscopy was performed under general anesthesia or conscious sedation (midazolam –0.2 mg/kg and meperidine – 1 mg/kg) using pediatric fibroscopy (Pentax FG24X and FG23H), after overnight fasting. Topic anesthesia and dimethicone were not used. Antral biopsies were taken from the antrum within about 2 cm of the pyloric channel for histology (2 fragments); the rapid urease test (2 fragments) and bacterial culture (2 fragments).

#### Histology.

The specimens were positioned on filter paper, fixed in 10% formaldehyde solution and stained by hematoxylin and eosin, and modified Giemsa. An expert pathologist characterized the presence of spiral bacteria in the mucosal layer or the surface of epithelial cells as a positive test.

#### Rapid urease test.

We used a homemade solution containing 1 ml of distilled water, 2 drops of 1% red phenol and 0.1g of urea. This solution was prepared by the endoscopist on the same day as the examination and was maintained at ambient temperature. The test was considered positive when the color changed from yellow to red and was observed for 24 hours.

#### Culture.

The fragment was inoculated in "Brain Heart Infusion" solution and maintained for 3 hours at 4^o^ C. Then we gently scraped the biopsy samples in "Brain Heart Infusion" agar containing 5% sheep blood and a selective medium with nalidixic acid (2.5 mg/ml), vancomycin (2.5 mg/ml) and amphotericin B (0.25 mg/ml). The plates were incubated at 37 ^o^C for 7 days under microaerophilic conditions. The bacterial culture was considered positive when small translucent colonies had spiral Gramnegative bacteria and were positive for the oxidase, catalase and urease tests.

### Non-invasive methods

Serological test: Prior to the endoscopic procedure, 5 ml of venous blood was drawn and serum was stored at -20 ^o^C for anti-*Helicobacter pylori* antibodies - IgG detection using the ELISA method (enzyme-linked immunosorbent assay - "Cobas Core II" – Roche, Hoffman La Roche Ltd., Switzerland). The results were considered positive when titers were greater than 7 U/ml, according to the manufacturer. ^13^Carbon-urea breath test: The test was performed with a commercial kit (ISOMED). Expired ^13^CO_2_ was collected in an appropriate tube and measured by mass spectrophotometer (Model 20/20 Europe Scientific Company, Manchester, UK). After overnight fasting, the patient ingested 4.2g of citric acid and aspartame dissolved in 200 ml of bottled water to delay gastric emptying, and after 10 minutes a basal sample of expired air was collected. ^13^C-urea (2 mg/kg – maximum 100 mg) dissolved in 50 ml of bottled water was ingested by patient and after 30 minutes a second sample was collected. The samples were sealed and dispatched for analysis within 3 months afterwards. The result was considered positive when the excretion rate was up to 3%.

### Statistic analysis

Sensitivity, specificity, and positive and negative predictive values^30^ were calculated for each method, considering any combination of 3 or more positive results out of the 5 methods as the gold standard. Cochran's G test^31^ was used to study agreement of positive and negative results, and McNemar's test^32^ to study disagreement between 2 methods. The rejection level for the null hypothesis was fixed at 0.05.

## RESULTS

The ages of the 47 patients ranged from 4y5m to 19y [mean 11y9m (SD 2y10m)], of whom 81% (38/47) were over 10 years old and 19% (9/47) under 10 years old; 57.4% (27/ 47) were female and 42.6% (20/47) were male. Abdominal pain was present in 98% (46/47), identified in the epigastric region in 85% and characterized as burning in 81%; vomiting was present in 70% and nocturnal pain in 64% of patients. A family history of peptic disease was present in 53% of patients and 4% presented upper digestive tract bleeding.

The endoscopic diagnosis was normal in 32% (15/47) and abnormal in 68% (32/47) and *Helicobacter pylori* infection was present in 62% (29/47). In 15 patients with a normal endoscopic examination, 73% (11/15) were *Helicobacter pylori*-negative and 27% (4/15) were *Helicobacter pylori*-positive. In 32 patients with an abnormal endoscopic examination, 78% (25/32) were *Helicobacter pylori*-positive and 22% (7/32) were *Helicobacter pylori*-negative (P < 0.05). Cases with abnormal endoscopy showed gastritis in 50% (16/32), esophagitis in 31% (10/32), duodenal ulcer in 16% (5/32) and duodenal erosions in 3% (1/ 32). *Helicobacter pylori* tested positive in 87.5% (14/16) of gastritis cases, in 50% (5/10) of esophagitis cases, in 100% of duodenal ulcers (5/5) and in 1 patient with duodenal erosions. Antral nodularity was observed in 72% (10/14) of endoscopic gastritis

Active chronic gastritis was observed in 72% (34/47) of patients: 79% (27/34) of them were *Helicobacter pylori*-positive and 21% (7/ 34) were *Helicobacter pylori*-negative patients. Despite the absence of inflammatory activity in 17% (8/47) of patients, 25% (2/8) of these were *Helicobacter pylori*-positive. A normal histological appearance occurred in 9% (5/47), all of whom were *Helicobacter pylori*-negative patients.

All the methods agreed in 61% (29/47) of patients, of which all were negative in 21% (10/47 – patients 1-10) and all positive in 40% (19/47 - patients 29–47) (Table 1). Disagreement occurred in 39% (Table 1): eight patients (17% - patients 21-28) presented 4 positive tests and 1 negative, seven (15%) patients (patients 11-17) presented 4 negative tests and one positive, two patients (4% - patients 19 and 20) presented three positive tests and 2 negative and one patient (2% - patient 18) presented 2 positive tests and other tests negative.

**Table 1:** Methods for diagnosing *Helicobacter pylori* infection

Culture	Patient	^13^C – urea breath test	Serology	Rapid urease test	Histology
01 - 10	-	-	-	-	-
11 - 13	+	-	-	-	-
14 - 15	-	+	-	-	-
16 - 17	-	-	+	-	-
18	+	+	-	-	-
19	+	+	+	-	-
20	+	-	+	+	-
21	+	-	+	+	+
22	+	+	+	-	+
23 - 24	-	+	+	+	+
25 - 28	+	+	+	+	-
29 - 47	+	+	+	+	+
TOTAL	31 (66%)	30 (64%)	31 (66%)	27 (57%)	23 (49%)

Cochran's G test; G calc = 13.52*; G critical = 9.49.

We observed that the greatest concordance occurred among invasive methods, 89.6% between histology and the rapid urease test and 87.5% between histology and culture. Of the non-invasive methods, serology presented the greatest concordance with invasive methods, 85.4% with the rapid urease test and 83.3% with histology (Table 2). The greatest sensitivity was achieved by the rapid urease test (100%), followed by histology (93.1%), serology (93.1%) and the ^13^carbon-urea breath test (93.1%) and lastly by culture (S = 79.3%). The highest specificity was obtained by histology (100%) and culture (100%), followed by the rapid urease test (84.2%), serology (78.9%) and ^13^carbon-urea breath test (78.9%) (Table 3).

**Table 2:** Disagreement analysis for the 5 methods

Tests	Agreement	Disagreement
Histology and Rapid Urease	89.6%	10.4%*
Histology and Culture	87.5%	12.5%
Rapid Urease and Serological	85.4%	14.6%
Histology and Serological	83.3%	16.7%
Histology and Urea Breath	83.3%	16.7%
Culture and Rapid Urease	81.2%	18.8%*
Rapid Urease and Urea Breath	81.2%	18.8%
Culture and Serological	79.2%	20.8%*
Serological and Urea Breath	79.1%	20.9%
Culture and Urea Breath	75%	25%*

*McNemar's Test*

* P < 0.05.

**Table 3:** Sensitivity, specificity, positive and negative predictive values, accuracy and confidence interval for the 5 methods.

Tests	Sensitivity (CI 95%)	Specificity (CI 95%)	Positive predictive value (%)	Negative predictive value (%)	Accuracy (%)
Rapid urease	100 (92.4 to 100)	84.2 (71.3 to 91.9)	91.2	100	93.7
Histology	93.1 (82.2 to 97.5)	100 (92.4 to 100)	100	90.5	95.8
Culture	79.3 (65.7 to 88.4)	100 (92.4 to 100)	100	76	87.5
Serology	93.1 (82.2 to 97.5)	78.9 (65.3 to 88.1)	87.8	88.2	87.5
^13^C-UBT	93.1 (82.2 to 97.5)	78.9 (65.3 to 88.1)	87.8	88.2	87.5

*^13^C-UBT = ^13^carbon-urea breath test.*

## DISCUSSION

The concordance of positive or negative results in 61% of cases was lower than observed in a similar study (87%),^26^ as was the concordance among positive results (40%) and negative results (21%), compared with positive (75%) and negative results (89.2%) in another study.^33^ This lower concordance is due to the occurrence of a high number of false results, as observed in Table 1: fifteen patients (32%) presented one discordant result, with this result being positive in 7 (patients 11-17) and negative in 8 (patients 21-28), which suggest these results may be false. But when more than one method presents discordance it is necessary to analyze carefully the methods and criteria used to classify the patient. Some authors use an association of methods as the gold standard,^20,28,29^ but this artifice can present failure, inducing errors in classifying the patient as infected or not infected. So it is very important to choose how many and which methods will be used as the gold standard. In this study we considered the positivity of 3 out of 5 evaluated methods, because the association of 2 methods could increase false positive results and 4 methods could increase false negative results.

Although advances in diagnostic methods have been achieved, accurate diagnosis has not yet been established. Invasive methods, on account of their high specificity, have been considered as the gold standard, but they present low sensitivity because they are biopsy-based methods.^1,2,32^ Determining which of them is the most accurate and available for routine use is harder than it seems. The association of histology and the rapid urease test, besides presenting better accuracy, is a relatively low cost one in comparison to other methods, and is practical. The combination of the two methods allows quick evaluation of the infectious status of patient^34,35^ (rapid urease test) and microscopic analysis of gastric mucosa (histology). As these are invasive methods, their combination is considered as the gold standard by a majority of authors,^20,21^ and it may be routinely indicated for investigation of patients submitted to endoscopy.

Bacterial culture presented the highest rate of false negative results, which decreased the sensitivity (79.3%) in comparison to other studies (86 to 100%).^36,37^ However, its specificity was high (100%): incontestable proof of the presence of bacteria.^9,20,38^ Difficulties in isolation and culturing, technical requirements and its relative low availability do not allow this method to be used as a single gold standard.

The rapid urease test and histology presented the best accuracy. The rapid urease test presented only 2 false positive results, demonstrating its high sensitivity (100%), as observed in another study,^26^ but disagreeing with others studies with low sensitivity in children.^25,34^ However, the specificity (84.2%) was lower due to false positive results. A homemade solution, easily made at low cost (estimated at US$ 0.01 per test), seems the best choice among the invasive methods. It is performed routinely in our service and allows quick evaluation of the infectious status of patients, which is done in the first 15 minutes of examination of all the *Helicobacter pylori*-positive patients. Histology showed high sensitivity (93%) as in other studies.^14,25^ The false negative results are expected in biopsy-based method, because it is possible to obtain a fragment without *Helicobacter pylori* due to patchy distribution of bacteria in gastric mucosa. It has been described as a high specificity method, as we observed (100%). All fragments were positioned on filter paper, because *Helicobacter pylori* is located deep in crypts and this positioning makes its investigation easier.^35^

The ^13^carbon-urea breath test presented a high occurrence of false results: 4 false positive and 2 false negative results. Despite the high sensitivity and specificity described for the method,^26,27,39^ our results (sensitivity = 93.1% and specificity = 78.9%) were lower than observed in other studies, especially by Thijs et al,^27^ who observed 100% sensitivity and specificity in a similar study among adults. The sensitivity, moreover, presents results closer to those described by Vandenplas et al,^26^ who evaluated pediatric patients. The low specificity, clearly lower than in other studies of children and adults, can perhaps be explained by oral urease^40^ or just possibly because of achlorhydria associated with other ureaseproducing bacteria or other Helicobacter spe-cies,^20,41^ but the small sample and lack of established cutoff for the Brazilian population, probably have a major influence on these results. Another difficulty was that the analysis was accomplished at a very distant location. Stored samples were dispatched by airmail, and although this is a practicable method, the long time elapsed after collection (3 months) could have influenced the results.

The results of serology showed 3 false positive and 2 false negative results. The high sensitivity (93%) observed is consistent with other pediatric studies in older children and adoles-cents,^14,26,33,42,43^ as was also the specificity (79%).^44,45^ However, these results must be evaluated carefully, considering that the age of patients can influenced serology results,^28,29^ because children's holdings of antibodies can be lower than for adults.^46-48^ These data make it difficult to standardize serology tests at pediatric ages. The results were better than reported from younger children (81% were older than 10 years). Although the ELISA method is an easily-performed, low cost and available method, the existence of different antigens in commercial kits^44^ can influence the accuracy of the method,^29^ with it being possible for discordance between sensitivity and specificity to oc-cur.^49,50^ These data indicate that is necessary to choose a non-commercial antigen that it is specific for each country or region^48^ and obtain validation of the method and the cutoff for the age and population being studied, due to differences in infection prevalence.^28,29^

In summary, our result indicated that bacterial culture alone could not be used as the gold standard due to its low sensitivity. Among the invasive methods, our results suggested that the association of the rapid urease test with histology constituted the best choice for confirming the diagnosis, due to its high concordance rate, the high sensitivity of the rapid urease test and high specificity of histology. And if discordance were to occur between these methods, the noninvasive method of serology could be used in older children, indicated because of its high concordance with invasive methods and higher specificity in comparison to the ^13^carbon-urea breath test. For better evaluation of the accuracy of non-invasive methods, the sample size would need to be increased.

## References

[1] Lindkvist P, Asrat D, Nilsson I (1996). Age at acquisition of *Helicobacter pylori* infection: comparison of a high and a low prevalence country. Scand J Infect Dis.

[2] Graham DY, Adam E, Reddy GT (1991). Seroepidemiology of *Helicobacter pylori* infection in India: comparison of developing and developed countries. Dig Dis Sci.

[3] Oliveira AMR, Queiroz DMM, Rocha GA, Mendes EN (1994). Seroprevalence of *Helicobacter pylori* infection in children of low socioeconomic level in Belo Horizonte, Brazil. Am J Gastroenterol.

[4] Simán JH, Forsgren A, Berglund G, Florén CH (1997). Association between *Helicobacter pylori* and gastric carcinoma in the city of Malmö, Sweden. Scand J Gastroenterol.

[5] Carvalho AST, Queiroz DMM, Mendes EN, Rocha GA, Penna FJ (1991). Diagnosis and distribution of *Helicobacter pylori* in the gastric mucosa of symptomatic children. Braz J Med Biol Res.

[6] Drumm B, O’Brien A, Cutz E, Sherman P (1987). Campylobacter pyloridis associated primary gastritis in children. Pediatrics.

[7] Oderda G, Dell'olio D, Morra I, Ansaldi N (1989). Campylobacter pylori gastritis: long term results of treatment with amoxicillin. Arch Dis Child.

[8] Hsu PI, Lai KH, Tseng HH (1997). Correlation of serum immunoglobulin G *Helicobacter pylori* antibody titers with histologic and endoscopic findings in patients with dyspepsia. J Clin Gastroenterol.

[9] Blecker U. (1996). *Helicobacter pylori* disease in childhood. Eur J Pediatr.

[10] Prieto G, Polanco I, Larrauri J, Rota L, Lama R, Carrasco S (1992). *Helicobacter pylori* infection in children: clinical, endoscopic, and histologic correlations. J Pediatr Gastroenterol Nutr.

[11] Blaser MJ (1990). Epidemiology of Campylobacter pylori infections. Rev Infect Dis.

[12] Dooley CP, Cohen H (1988). The clinical significance of Campylobacter pylori. Ann Intern Med.

[13] Peterson WL (1991). *Helicobacter pylori* and peptic ulcer disease. N Engl J Med.

[14] Killbridge PM, Dahms BB, Czinn SJ (1988). Campylobacter pylori associated gastritis and peptic ulcer disease in children. Am J Dis Child.

[15] Lin JT, Wang LY, Wang JT, Wang TH, Chen CJ (1995). Ecological study of association between *Helicobacter pylori* infection and gastric cancer in Taiwan. Dig Dis Sci.

[16] Wotherspoon AC, Ortiz-Hidalgo C, Falzon MR, Isaacson PG (1991). *Helicobacter pylori*-associated gastritis and primary B-cell gastric lymphoma. Lancet.

[17] Horstmann M, Erttmann R, Winkler H (1994). Relapse of MALT lymphoma associated with *Helicobacter pylori* after antibiotic treatment. Lancet.

[18] Barabino A, Dufour C, Marino CE, Claudiani F, De Alessandri A (1999). Unexplained refractory iron-deficiency anemia associated with *Helicobacter pylori* gastric infection in children: Further clinical evidence. J Pediatr Gastroenterol Nutr.

[19] Marignani M, Angeletti S, Bordi C (1997). Reversal of long-standing iron deficiency anemia after eradication of *Helicobacter pylori* infection. Scand J Gastroenterol.

[20] Mégraud F (1996). Advantages and disadvantages of current diagnostic tests for the detection of *Helicobacter pylori*. Scand J Gastroenterol.

[21] Morris A, Ali MR, Brown P, Lane M, Patton K (1989). Campylobacter pylori infection in biopsy specimens of gastric antrum: laboratory diagnosis and estimation of sampling error. J Clin Pathol.

[22] Fennerty MB (1994). Helicobacter pylori. Arch Intern Med.

[23] Cutler AF, Haustad S, Ma CK, Blaser MJ, Perez-Perez GI, Schubert TT (1995). Accuracy of invasive and non-invasive tests to diagnose *Helicobacter pylori* infection. Gastroenterology.

[24] Hodgson MI, Pantoja H, Latorre JJ (1995). *Helicobacter pylori*-associated gastroduodenal disease in symptomatic Chilean children: diagnostic value of serological assay. J Pediatr Gastroenterol Nutr.

[25] Heldenberg D, Wagner Y, Heldenberg E (1995). The role of *Helicobacter pylori* in children with recurrent abdominal pain. Am J Gastroenterol.

[26] Vandenplas Y, Blecker U, Devreker T (1992). Contribution of the 13 C-urea breath test to the detection of *Helicobacter pylori* gastritis in children. Pediatrics.

[27] Thijs JC, Van Zwet AA, Thijs WJ (1996). Diagnostic tests for *Helicobacter pylori*: a prospective evaluation of their accuracy, without selecting a single test as the gold standard. Am J Gastroenterol.

[28] Feldman RA, Evans SJW (1995). Accuracy of diagnostic methods used for epidemiological studies of *Helicobacter pylori*. Aliment Pharmacol Ther.

[29] Bodhidatta L, Hoge CW, Churnratanakul S (1993). Diagnosis of *Helicobacter pylori* infection in a developing country: comparison of two ELISAs and a seroprevalence study. J Infect Dis.

[30] Gallen RS, Gambino SR (1975). Beyond normality: the predictive value and efficiency of medical diagnoses.

[31] Siegel S, Casttelan NJ (1988). Non-parametric statistics.

[32] Remington RD, Schork MA (1970). Statistics with applications to biological and health sciences.

[33] Chong SKF, Lou Q, Asnicar MA (1995). *Helicobacter pylori* infection in recurrent abdominal pain in childhood: Comparison of diagnostic tests and therapy. Pediatrics.

[34] Elitsur Y, Hill I, Lichtman SN, Rosenberg AJ (1998). Prospective comparison of rapid urease tests (Pyloritek, CLOTest) for the diagnosis of *Helicobacter pylori* infection in symptomatic children: a pediatric multicenter study. Am J Gastroenterol.

[35] Zaitoun AM (1993). Histology compared with chemical testing for urease for rapid detection of *Helicobacter pylori* in gastric biopsy specimens. J Clin Pathol.

[36] Veldhuyzen SJO, Tytgat K, deGara CJ (1991). A prospective comparison of symptoms and five diagnostic tests in patients with *Helicobacter pylori* positive and negative dyspepsia. Eur J Gastroenterol Hepatol.

[37] Marshall BJ, Guerrant RL, Plankey MW (1988). Comparison of ^
13
^C-urea breath test, microbiology and histology for the diagnosis of Campylobacter pylori. (abstract) Gastroenterology.

[38] Van Zwet AA, Thijs JC, Kooistra-Smid AMD, Schirm J, Snijder JAM (1993). Sensitivity of culture compared with that of polymerase chain reaction for detection of *Helicobacter pylori* from antral biopsy samples. J Clin Microbiol.

[39] Rowland M, Lambert I, Gormally S (1997). Carbon 13-labeled urea breath test for the diagnosis of *Helicobacter pylori* infection in children. J Pediatr.

[40] Raju GS, Smith MJ, Morton D, Bardhan KD (1994). Mini-dose (1-µCi) ^
14
^C-urea breath test for detection of *Helicobacter pylori*. Am J Gastroenterol.

[41] Hamlet AK, Erlandsson KIM, Olbe L, Svennerholm AM, Backman VEM, Pettersson AB (1995). A simple, rapid, and highly reliable capsule-based ^
14
^C urea breath test for diagnosis of *Helicobacter pylori* infection. Scand J Gastroenterol.

[42] Blecker U, Lanciers S, Hauser B, Vandenplas Y (1993). Diagnosis of *Helicobacter pylori* infection in adults and children using the Malakit *Helicobacter pylori*, a commercially available enzyme-linked immunosorbent assay. J Clin Microbiol.

[43] Raymond J, Kalach N, Bergeret M, Barbet JP, Benhamou PH, Gendrel D (1996). Evaluation of a serological test for diagnosis of *Helicobacter pylori* infection in children. Eur J Clin Microbiol Infect Dis.

[44] Westblom TU, Madan E, Gudipati S, Midkiff BR, Czinn SJ (1992). Diagnosis of *Helicobacter pylori* infection in adult and pediatric patients by using Pyloriset, a rapid latex agglutination test. J Clin Microbiol.

[45] Goossens H, Glupczynski Y, Burette A, Van Den Boore C, Butzler JP (1992). Evaluation of a commercially available second-generation immunoglobulin G enzyme immunoassay for detection of *Helicobacter pylori* infection. J Clin Microbiol.

[46] Russell RG, Wassermann SS, O’Donnohue JM (1993). Serologic response to *Helicobacter pylori* among children and teenagers in northern Chile. Am J Trop Med Hyg.

[47] Carmolinga-Ponce M, Torres J, Perez-Perez G (1998). Validation of a serologic test for the diagnosis of *Helicobacter pylori* infection and the immune response to urease and CagA in children. Am J Gastroenterol.

[48] Khanna B, Cutler A, Israel NE (1998). Use caution with serologic testing for *Helicobacter pylori* infection in children. J Infect Dis.

[49] Hoek FJ, Noach LA, Raws EAJ, Tytgat GNJ (1992). Evaluation of the performance of commercial test kits for detection of *Helicobacter pylori* antibodies in serum. J Clin Microbiol.

[50] Perez-Perez GI, Taylor DN, Bodhidatta L (1990). Seroprevalence of *Helicobacter pylori* infections in Thailand. J Infect Dis.

